# Depression and mental wellbeing in people affected by leprosy in southern Nepal

**DOI:** 10.1080/16549716.2020.1815275

**Published:** 2020-10-01

**Authors:** Marloes M. A. R. van Dorst, Wiebrich J. van Netten, Mitzi M. Waltz, Basu D. Pandey, Ramesh Choudhary, Wim H. van Brakel

**Affiliations:** a Athena Institute, VU University Amsterdam, Amsterdam, Netherlands; b Sukra Raj Tropical and Infectious Disease Hospital, Ministry of Health and Population, Kathmandu, Nepal; c Department Community, Lalgadh Leprosy Hospital & Services Centre, Janakpur, Nepal; d NLR, Amsterdam, Netherlands

**Keywords:** Leprosy, depression, mental wellbeing, stigma, Nepal

## Abstract

**Background:**

Leprosy, a leading cause of disability, remains endemic in southern Nepal. Alongside physical impairment and stigmatization, many people affected by leprosy suffer from mental health problems.

**Objectives:**

This study had two objectives: (a) Establishing a baseline level of mental wellbeing and depression among people affected by leprosy in southern Nepal, and (b) Examining factors that influence mental wellbeing and depression in this target group.

**Methods:**

A cross-sectional survey was conducted using three interview-administered questionnaires measuring level of depression (PHQ-9), mental wellbeing status (WEMWBS) and level of stigma (5-QSI-AP). Random clustering sampling was used to include leprosy-affected people from Self Help Groups (SHGs) and the reference group was matched based on socio-demographic characteristics. All participants were adults with no additional major morbidities. A sample of 142 persons affected by leprosy and 54 community controls were included.

**Results:**

People affected by leprosy participating in SHGs had a significantly lower level of mental wellbeing and higher level of depression than the general population. Both mental wellbeing and depression were influenced by gender and the level of stigma. In addition, the level of depression was associated with the disability grade of leprosy-affected people.

**Conclusion:**

Leprosy-affected people need mental health-care interventions at different organizational levels, with attention to identifying individuals at increased risk for mental health problems or with additional needs. These findings highlight the demand for further research on specific interventions to improve the mental health of leprosy-affected people.

## Background

Neglected Tropical Diseases (NTDs) are a diverse group of diseases caused by parasitic, viral and bacterial infections that prevail in tropical conditions. The World Health Organization (WHO) has selected 20 NTDs as a focus for elimination, including leprosy [1]. Leprosy is caused by *Mycobacterium leprae* and typically affects the peripheral nerves, eyes, skin and mucosa of the upper respiratory tract [2]. This can cause irreversible nerve damage resulting in disabilities and secondary impairments. Leprosy remains an important cause of preventable disability throughout the world with 208,619 new cases of leprosy being diagnosed globally in 2018 [3]. More than 70% of these new cases are found in South Asia [3]. In the southern part of Nepal, the Terai region and the neighbouring north-eastern part of India, leprosy is still highly endemic and in Nepal alone 3,775 new cases of leprosy were reported in 2018 [4].

Alongside experiencing physical impairment, many people affected by leprosy experience stigmatization. Several studies have reported that people affected by leprosy often experience rejection, hate, insults and unsympathetic reactions [5–8]. In western Nepal, high levels of perceived stigma among leprosy-affected people were reported [9]. Stigmatization can result in mental health problems and amongst leprosy-affected people reduced mental wellbeing is frequently reported [10–13]. A study in Bangladesh demonstrated that leprosy strongly influences the mental wellbeing of those affected by this disease and in Ethiopia, a study reported that people affected by leprosy had a sevenfold increased risk of mental disorders in comparison to those affected by other skin diseases [12,13].

Several studies have reported stigmatization and mental health problems in leprosy-affected people [10–13]. However, little is known about the factors associated with depression and mental wellbeing in leprosy-affected people in southern Nepal. This study had two main aims: (a) Establishing a baseline of the mental wellbeing status and level of depression in the leprosy-affected population of southern Nepal, and (b) to examining factors that influence mental wellbeing and depression amongst people affected by leprosy in southern Nepal. This study highlights the importance of psychosocial aspects of leprosy in addition to its physical impacts and suggests directions for interventions and future research to increase the wellbeing of leprosy-affected people.

## Methods

### Sample population

For this research, the study population was comprised of people affected by leprosy in southern Nepal. Since the catchment area of LLHSC is extensive and many communities are difficult to reach, data were collected from people affected by leprosy who participated in Self Help Groups (SHGs). These groups meet regularly and are part of the outpatient services of Lalgadh Leprosy Hospital & Services Centre (LLHSC) located in southern Nepal. All people diagnosed with leprosy are invited to join these groups regardless of the severity of their disability or mental health status. Alongside self-care to improve their physical conditions, SHG participants also benefit from micro-credit schemes.

### Sample size and sampling method

Sample size was calculated using EpiCalc2000 (Brixton Health) and was based on the prevalence found by Risal *et al*. in Nepalese adults [14]. A minimal sample size of 80 leprosy-affected participants was needed to obtain a prevalence estimate of depression with a precision of ± 5% and a confidence level of 95%. For the reference group, a minimum of 50 was needed. Random cluster sampling of SHGs was used to include leprosy-affected people. We followed a predetermined visiting schedule made by the community staff of LLHSC and included all eligible members of the SHGs visited. To obtain reference data for comparison of the level of depression and mental wellbeing status, people from the general population were included in a reference group using purposive sampling. They were selected from the villages visited for the SHG meetings and were matched with the leprosy-affected group based on socio-demographic information (e.g. sex, age, caste).

### Inclusion and exclusion criteria

People were included in the leprosy-affected group of this study if they were affected by leprosy, were over 18 years of age, and had participated in a SHG for more than 3 months. People were excluded from participation in the study if they had another major morbidity (e.g. polio, HIV/AIDS) or were not resident in the LLHSC catchment area. The reference group had the same inclusion and exclusion criteria, except its members were not affected by leprosy and did not participate in SHGs.

### Data collection

The data collection instruments included a socio-demographic data questionnaire and three questionnaires related to mental well-being, depression and stigma. The months since a leprosy-affected person had been diagnosed was reported and the level of impairment of leprosy patients was assessed using the WHO three-grade system. A disability grade of 0 indicates no disability (impairment), grade 1 is sensory impairment and grade 2 indicates visible disfigurement, such as contractures of digits, wounds, loss of digits and blindness [15]. Information regarding socio-demographics, mental wellbeing and depression was obtained from all participants (n = 196). Data on leprosy-related characteristics (e.g. level of impairment) was gathered from all leprosy-affected people (n = 142) and the stigma survey was only conducted in a subset of leprosy-affected people (n = 86).

We used the Patient Health Questionnaire (PHQ-9), an interview-administered questionnaire based on the fourth version of the Diagnostic and Statistical Manual of Mental Disorders (*DSM*), to measure the level of depression [16]. Each of the nine *DSM-IV* criteria of depression is scored from 0 (not at all) to 3 (nearly every day) in the past 2 weeks, giving a total score ranging from 0 to 27. Based on previous literature, a score equal to or higher than 10 was considered to indicate depression [17,18]. The PHQ-9 questionnaire has a good internal consistency (Cronbach’s *a* = 0.835), a content validity that is based on the *DSM-IV*, and a solid one-month test–retest reliability of *r* = 0.875 [19,20].

To measure mental wellbeing, the Warwick–Edinburgh Mental Wellbeing Scale (WEMWBS) was used. This scale was developed to monitor mental wellbeing in the general population and includes 14 items regarding positive attributes of mental wellbeing [21]. Responses range from 1 (none of the time) to 5 (all of the time), giving a total score ranging from 0 to 70. Based on previous research a score of 45 or lower on the WEMWBS was considered to represent poor mental wellbeing [22]. Cross-cultural evaluation showed that the WEMWBS has a good internal consistency (Cronbach’s *a* = 0.91) and a high test–retest reliability at 1 week (*r* = 0.83) [23]. This was supported by studies that demonstrated the validity and reliability of this questionnaire in different contexts [24,25]. For both the PHQ-9 and the WEMWBS, good cultural validity in Southern Nepal was established by Dijkstra *et al*., 2018 (available from NTD.org).

To determine the level of stigma, the 5-Question Stigma Indicator-Affected Persons (5-QSI-AP) was used. The 5-QSI-AP is mentioned in the ‘suggested actions’ section of the *Global Leprosy Strategy 2016–2020 Monitoring & Evaluation Manual* as an easy-to-use questionnaire for stigma assessment [26]. This questionnaire is based on the EMIC stigma scale, which is a commonly used stigma questionnaire [27]. Each of the five questions of this questionnaire can be scored 0 (never or I do not know), 1 (sometimes) or 2 (often/usually), giving a total score ranging from 0 to 10.

### Data analysis

All scores were entered in EpiInfo version 7.2 (CDC) and exported to SPSS (IBM SPSS Statistics 25) for further analysis. Descriptive statistics were used for analysis of the socio-demographic and leprosy-associated characteristics. To compare continuous variables between two groups, a Student’s t-test was applied for parametric data. For the analysis of categorical variables, the Pearson’s Chi-square test was executed. In case one or more relevant cells did not contain any observations, Haldane-Anscombe correction was applied by adding 0.5 to each of the relevant cells. Subsequently, the statistical testing was performed using these adjusted cell counts. To examine factors associated with mean depression and mental wellbeing scores, bootstrapped regression was performed, as bootstrapping corrects for non-normality by making no assumptions about the distribution of the data. Bootstrapped univariate linear regression was performed on all variables that had a minimum of 20 observations in each category. Subsequently, multivariate linear regression was performed including all variables that were found to be significant in the univariate analyses. Finally, Cronbach’s alpha was calculated in SPSS to validate the 5-QSI-AP stigma scale. For all statistical tests a p-value ≤0.05 was considered significant.

### Ethical considerations

The data were collected in May and June 2018 after ethical approval was obtained (Reg. no. 79/2018) from the Nepal Health Research Council (NHRC). Before any data were collected, participants were informed about the study and informed consent was obtained.

## Results

### Characteristics study population

Table 1 compares the characteristics of people affected by leprosy and the reference group. In total, 196 persons were included, of which 142 people were affected by leprosy and 54 were community controls. The former had a mean age of 56.8, with a roughly equal distribution among the sexes. The reference group had a mean age of 61.1 and included more women (63.0%) than men. In both groups the majority of people lived in a rural area, were Hindu, belonged to the lowest caste and were illiterate and currently married. Agricultural work and household work were the two most common forms of occupation in both groups. The average length of time since respondents had been diagnosed with leprosy was 170 months (more than 14 years) and more than 16% of the leprosy-affected people had a disability grade of 2, indicating visible disfigurements.

**Table 1.** Characteristics of people affected by leprosy and the reference group in South-East Nepal.

	Leprosy- affected n (%) N = 142	Reference n (%) N = 54
Mean age (SD)	56.8 (13.54)	61.1 (15.193)
Gender, male	73 (51.4)	20 (37.0)
Living in rural area	127 (89.4)	50 (92.6)
District**		
Dhanusha	58 (40.8)	33 (61.1)
Mahottari	63 (44.4)	10 (18.5)
Sarlahi	21 (14.8)	11 (20.4)
Religion, Hinduism	135 (95.1)	53 (98.1)
Caste, low	109 (76.8)	45 (83.3)
Education, illiterate	119 (83.8)	49 (90.7)
Marital status, married	110 (77.5)	41 (75.9)
Occupation		
Own business	15 (10.6)	6 (11.1)
Employed	10 (7.0)	0
Unemployed	6 (4.2)	0
Agricultural labor	57 (40.1)	17 (31.5)
Household work	54 (38.0)	31 (57.4)
Disability grade, grade 2	23 (16.2)	NA
How many months ago diagnosed (SD)	170.3 (137.4)	NA

For categorical variables Pearson’s Chi-square test was performed and for continuous normally distributed variables (age) a student’s t-test was performed. A *p*-value ≤ 0.05 was considered significant. *p ≤ 0.05, **p ≤ 0.01, ***p ≤ 0.001. *SD* = Standard deviation. N = total number of subjects in that group. n = number of subjects within that category.

### Level of depression and mental wellbeing status

**Figure 1.**
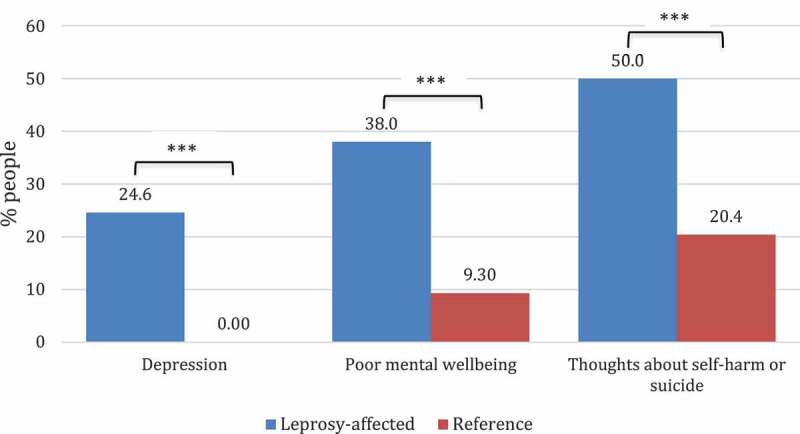
Prevalence of depression (total PHQ-9 score ≥ 10), poor mental wellbeing (total WEMWBS score ≤ 45) and thoughts about self-harm or suicide (score PHQ-9 question 9 ≥ 1) in the leprosy affected (n = 142) and reference group (n = 54).

The mean total PHQ-9 score was significantly higher in the leprosy-affected group than in the reference group (*p* < 0.001), with a mean of 7.54 (95% CI 6.63–8.46) among people affected by leprosy and 3.61 (95% CI 2.95–4.27) in the reference group. The frequency of depression amongst people affected by leprosy was 105, corresponding to 24.6% (95% CI 16.9–31.1) of the leprosy-affected people in this study population. In contrast, no one in the reference group had a PHQ-9 score higher than or equal to 10, which would indicate depression. These results demonstrate a significantly higher frequency of depression in leprosy-affected people compared to the reference group (*p* < 0.001) (Figure 1).

Furthermore, the mean total WEMWBS score was significantly lower in leprosy-affected people (49.0, 95% CI: 47.0–50.9) compared to the reference group (58.3, 95% CI 55.9–60.7) (*p* < 0.001). The frequency of people with poor mental wellbeing was 54 in the leprosy-affected people, which corresponds to 38.0% (95% CI: 30.1–45.9) and 5 people in the reference group, which corresponds to 9.3% (95% CI 1.27–16.7). These results show that the frequency of poor mental wellbeing was significantly higher in the leprosy-affected group compared to the reference group (*p* < 0.001) and that the risk of poor mental wellbeing was six times higher in people affected by leprosy than in the reference group (odds ratio of 6.02 (95% CI 2.26–16.12), *p* < 0.001) (Figure 1).

Haldane Anscombe correction was applied if cells contained less than 1 observation before Pearson’s Chi-square was performed. A p-value ≤ 0.05 was considered significant *p ≤ 0.05, **p ≤ 0.01, ***p ≤ 0.001.

Finally, the frequency of thoughts about self-harm or suicide was significantly higher in leprosy-affected people (71 people, corresponding to 50%) compared to the reference group (11 people, corresponding to 20.4%) (*p* < 0.001). Of the leprosy-affected people with these thoughts, 3 people indicated to have these thoughts more than half the days in the past 2 weeks (score of 2) and 8 had them nearly every day in this period (score of 3), whereas none of the people of the reference group of with these thoughts had these thoughts more than several days in the past 2 weeks (score of 1).

### Factors influencing depression and mental wellbeing

Since the stigma survey (5-QSI-AP) was only administered to a subset of people affected by leprosy, regression analysis was performed on this smaller group of leprosy-affected people (*n* = 86). The socio-demographic and leprosy-associated characteristics of this subset resembled those of the larger sample of leprosy-affected people that was presented earlier (*n* = 142) (Table S1). In addition, only risk factors with a sufficient distinctive power and at least 20 observations per category were included in the bootstrapped regression analysis.

The results showed that the mean total PHQ-9 score was significantly higher if the participant was a woman (*p* = 0.023) and had a disability grade of 2 (*p* = 0.023). In addition, the mean total 5-QSI-AP score was positively associated with the levels or depression, with a regression coefficient of 1.36 (95% CI 0.78–2.01, *p* = 0.011). The mean total PHQ-9 score was not significantly affected by the other variables, which included age, caste and marital status (Table 2). The multivariate regression showed that mean total 5-QSI-AP score (β = 1.110, p = 0.011), gender (β = 2.619, p = 0.023) and disability grade (β = 3.763, p = 0.046) were all independently associated with the mean total PHQ-9 score and explained 37.0% of the score (R square = 0.370) (Table 3).

**Table 2.** Associations between risk factors and the total mean PHQ-9 score or total mean WEMWBS score among leprosy-affected people in southern Nepal (N = 86).

	n	Mean total PHQ-9 score (95% CI)	p-value	Mean total WEMWBS score (95% CI)	p-value
Age					
≤ 50 years	25	6.9 (5.3-8.6)	0.885	48.0 (42.3-53.7)	0.690
> 50 years	61	7.2 (6.0-8.2)		49.1 (45.5-53.8)	
Gender					
Male	37	5.7 (4.8-6.9)	0.023*	53.8 (49.9-57.7)	0.011*
Female	49	8.1 (7.0-9.2)		45.0 (41.8-49.1)	
Disability Grade					
0 & 1	66	6.0 (5.0-6.6)	0.023*	50.3 (47.6-53.2)	0.069
2	20	10.7 (8.3-12.7)		43.8 (36.9-50.6)	
Caste					
Middle or High	23	5.5 (4.2-6.7)	0.069	52.1 (46.2-58.2)	0.069
Low	63	7.7 (6.1-8.7)		47.6 (43.8-52.2)	
Marital status					
Married	65	6.4 (5.5-7.2)	0.103	50.5 (47.4-53.2)	0.069
Not married	21	9.1 (6.6-11.7)		43.6 (35.6-52.0)	
Mean total 5-QSI-AP score	86	1.36 (0.78-2.01)^1^	0.011*	-2.61 (-4.16; -0.53)^1^	0.011*

1. Regression coefficient (β) and 95% CI of mean total 5-QSI-AP score. Bootstrapped univariate regression was performed. A p-value ≤ 0.05 was considered significant, *p ≤ 0.05, **p ≤ 0.01, ***p ≤ 0.001. n = number of subjects within that category. 95% CI = 95% confidence interval, PHQ-9 = Patient Health Questionnaire, WEMWBS = Warwick-Edinburgh Mental Wellbeing Scale, 5-QSI-AP = 5-Question Stigma Indicator-Affected Persons.

**Table 3.** Multivariate regression analysis risk factors and the total mean PHQ-9 score or total mean WEMWBS score among leprosy-affected people in southern Nepal (N = 86).

	Regression coefficient (β)	Standard error	p-value
PHQ-9			
Constant	*2.817*	*0.919*	*0.011*
Mean total 5-QSI-AP score	1.110	0.381	0.011*
Gender			
Male	Reference		
Female	2.619	0.936	0.023*
Disability grade			
0 & 1	Reference		
2	3.763	1.577	0.046*
WEMWBS			
Constant	*57.667*	*1.822*	*0.011*
Mean 5-QSI-AP score	-2.477	0.679	0.011*
Gender			
Male	Reference		
Female	-8.168	2.155	0.023*

Bootstrapped multivariate regression was performed with the risk factors identified in the bootstrapped univariate regression (table 2). The model of PHQ-9 explains 37.0% of the score (R square = 0.370) and the WEMWBS model 28.8% (R square = 0.288).

A p-value ≤ 0.05 was considered significant, *p ≤ 0.05, **p ≤ 0.01, ***p ≤ 0.001. n = number of subjects within that category. 95% CI = 95% confidence interval, PHQ-9 = Patient Health Questionnaire, WEMWBS = Warwick-Edinburgh Mental Wellbeing Scale, 5-QSI-AP = 5-Question Stigma Indicator-Affected Persons.

Univariate regression analysis showed that the mean total WEMWBS score was significantly lower if the participant was a woman (*p* < 0.011) and that the mean total WEMWBS score was negatively associated with the mean total 5-QSI-AP score, with a regression coefficient of −2.61 (95% CI −4.16; −0.53, *p* = 0.011). The mean total WEMWBS score was not significantly affected by age, caste and marital status (Table 2). Both gender (β = −8.168, *p* = 0.023) and mean total 5-QSI-AP score (β = −2.477, *p* = 0.011) remained significantly associated with the mean total WEMWBS score in the multivariate analysis and explained 28.8% of the score (R square = 0.288) (Table 3).

### Stigma

The **significant association**s between the **total mean 5-QSI-AP in leprosy-affected people** and **the total mean PHQ-9 score and total mean WEMWBS score support**ed **the construct validity of this scale**. Additional analysis was performed in order to examine the validity of the 5-QSI-AP scale in this population, showing that this questionnaire had a Cronbach’s alpha of 0.74. Furthermore, the results showed that none of the questions had missing values except for the fourth question. This question was back-translated from Maithili as: ‘Would leprosy cause a problem for a person to get married or in an existing marriage?’ and 25% of the sample did not answer this question. Finally, more than 15% of the sample scored the minimum score of zero (no stigma), which indicates a floor effect.

## Discussion

### Level of depression and mental wellbeing status

Our study showed that people affected by leprosy had a higher level of depression than people from the reference group. The mean depression score in leprosy-affected people was significantly higher compared to the reference group (respectively 7.54 *vs* 3.61, *p < *0.001), which is in line with the study of Tsutsumi *et al*., reporting higher mean depression scores in people affected by leprosy compared to the general population in Bangladesh [13]. Furthermore, the prevalence of depression among people affected by leprosy was significantly higher than in the reference group (24.6% *vs* 0%). The prevalence of depression among leprosy-affected people found in this study is in line with prevalence rates reported in literature, which strongly vary and range from 10% to 76% [10,28–32]. The finding that none of the people in the reference group had a depression, differed from the study of Risal *et al*. reporting an age- and gender-adjusted prevalence of depression of 4.2% in Nepalese adults [14]. The questionnaire used in the study of Risal *et al*., however, differed from that in our study. Moreover, the reference group in our study is not a representative sample of Nepalese adults, since the participants were selected to match the socio-demographic characteristics of the leprosy-affected people included in this study.

Furthermore, we showed that the prevalence of thoughts about suicide or self-harm was significantly higher (p < 0.001) among people affected by leprosy (50%) compared to the reference group (20%). Although the absolute prevalence we found is much higher, the higher prevalence in leprosy-affected people is in line with the study of Leekassa *et al*., who reported more suicidal ideation in people affected by leprosy (18.5%) compared to people with other skin conditions (6.3%) in Ethiopia [11]. The higher prevalence found in our study in both the reference and leprosy-affected group might be caused by cultural differences regarding self-harm and suicidal thoughts but for this specific population, this has not (yet) been described in the literature. Moreover, this study reports thoughts about both self-harm and suicide, whereas most studies only report suicidal thoughts including the study by Leekassa et al. [11].

In line with the above, we found that the mean mental wellbeing score was significantly lower in the leprosy-affected group than in the reference group (respectively 49.0 *vs* 58.3, *p* < 0.001), which indicates that leprosy-affected people experience poorer mental wellbeing. Studies among healthy adults have reported a mean total WEMWBS score of 48.1 in Pakistan and around 50 in the UK [21,23,33,34]. To our knowledge, no other studies among leprosy-affected people have been performed using this measure. Therefore, studies in leprosy-endemic countries and among leprosy-affected people using the WEMWBS scale are needed to establish whether the lower mental wellbeing among people affected by leprosy is a universal or population-specific phenomenon.

Next, the results showed that the prevalence of poor mental wellbeing was significantly higher in people affected by leprosy than in the reference group (38% vs 9.3%, *p* < 0.001) and that people affected by leprosy had a six-fold increased risk of having poor mental wellbeing (*OR* = 6.02 (95% CI 2.26–16.12), *p* < 0.001). These findings are in line with the study of Leekassa *et al*., reporting a higher level of mental distress amongst people affected by leprosy in Ethiopia. The Ethiopian study reported a prevalence of mental distress of 52% amongst leprosy-affected people compared to 7.9% in those with other skin conditions, which corresponds to a seven-fold increased risk of mental distress (OR = 7.14, 95% CI 4.15–12.35) in leprosy-affected people compared to people with other skin conditions [11].

The high prevalence of depression and poor mental wellbeing in the leprosy-affected people in our sample highlights the impact of leprosy on mental health. The leprosy-affected people in our sample population participate in a SHG, which is supported by staff of LLHSC and aims to improve the physical condition, social participation and socioeconomic position of its participants. It is known that SHGs have a strong positive impact on the group members, in terms of reducing the effect of stigma and improving self-esteem [35–38]. The fact that despite such activities, mental wellbeing is still poor in more than one-third of the participants and that depression is prevalent indicates that separate attention is needed to treat depression and improve mental wellbeing in such programmes.

### Factors associated with depression and mental wellbeing

Gender, disability grade and the level of stigma were independently associated with the level of depression in our study and the mental wellbeing status was independently affected by both gender and the level of stigma. We found that women had a higher level of depression and poorer mental wellbeing and that leprosy-affected people with visible disfigurements (disability grade 2) had a higher level of depression. The level of stigma was positively associated with the depression score and negatively associated with the mental wellbeing score, indicating a higher level of depression and poorer mental wellbeing in people that experience stigmatization.

The finding that, on average, women had a higher level of depression and poorer mental wellbeing is in line with our expectations and observations. Gender inequalities in combination with leprosy are often described in the literature and is referred to as facing ‘double jeopardy’ [38,39]. This is in line with a recent review describing the inferior position of women with leprosy, who often face more stigmatization, concealment, treatment delay, difficulties with marriage and social rejection resulting in lower quality of life and higher mental burden [40]. These problems in marital life and sexuality among women in South-East Nepal were also reported by Van ‘t Noordende et al. (2016). Finally, the study of Van Netten *et al*. revealed that women in general in southern Nepal are dependent on their husbands, or family-in-law if widowed, which puts women in a vulnerable position if she is affected by leprosy (article in preparation).

The association between disability grade and levels of depression are in line with previous studies.

Leekassa *et al*. showed that a high disability score was significantly associated with mental distress (*OR* = 3.96) and Tsutsumi *et al*. reported that the quality of life and general mental health of leprosy-affected people tended to be worse for people with disfigurement than for those without [11,39]. The higher level of depression in disfigured people affected by leprosy might be caused by the increased participation restrictions experienced by these people. It has been reported that participation restrictions among leprosy-affected people greatly impact social life and can seriously affect mental wellbeing [9,27,35].

Our findings show that the level of stigma is independently associated with the level of depression and mental wellbeing status among leprosy-affected people. These results are in line with the study of Tsutsumi *et al*. in Bangladesh, who reported that depression scores were higher in people with high level of self-perceived stigma compared to people with low levels of self-perceived stigma [13]. Another study by Tsutsumi *et al*. revealed that perceived stigma significantly affected all aspects of quality of life, including the psychological subdomain [39]. Finally, the results are in line with the review of van Brakel [6], which states that stigma affects people psychologically: it can cause or aggravate psychiatric morbidity and contributes to lower self-esteem.

### Stigma in leprosy-affected people

To validate the stigma scale used in this study, the 5-Question Stigma Indicator-Affected Persons (5-QSI-AP), we determined the internal consistency, missing values and floor and ceiling effects. We found that this questionnaire has a good internal consistency (Cronbach’s *a* = 0.74), which is high considering that the 5-QSI-AP has only 5 items. There were no missing values except for question 4 (‘Would leprosy cause a problem for a person to get married on in an existing marriage?’). These missing values might be due to the use of confusing language or could be caused by the fact that a substantial part of the sample population was widowed and therefore thought they were not able to answer this question. Finally, the results show that more than 15% of people scored the minimum score of zero, which indicates a floor effect. However, a high sensitivity near the zero level is not needed for this scale, since a low score means that the person does not experience stigma, and thus no interventions would be needed anyway.

### Limitations

One limitation of this study is that the participants affected by leprosy were members of SHGs. Since the SHG programme is likely to have a positive influence on participant’s mental health and wellbeing, leprosy-affected SHGs participants may have lower levels of depression and better mental wellbeing than people affected by leprosy who do not participate in SHGs. Inclusion of leprosy-affected people that are members of SHGs might therefore have caused an underestimation of the level of depression in leprosy-affected people. On the other hand, by including people who participate in SHGs, the level of depression and poor mental wellbeing in this study also might be an overestimation, since leprosy-affected persons who are in need of help are more likely to participate in these SHGs. Next, socio-demographic characteristics of the leprosy-affected people in this study were homogenous, which limited the possibilities to identify factors associated with depression and poor mental wellbeing. It is therefore likely that in addition to the variables found in this study, other factors might play a role in depression and poor mental wellbeing. Another limitation is that there are considerable differences in setting, culture, ethnicity, local language and environment among districts in Nepal. The results of this study are therefore not necessarily representative of the whole of Nepal, and should not be generalised without confirmation.

### Implications

This study shows that the psychosocial aspects of leprosy are as important as the physical aspects. The findings suggest that mental health interventions are needed for many South Nepali leprosy-affected people, including persons who already participate in SHGs. These interventions should be developed at different organizational levels. First of all, it might be helpful to offer group counselling to members of SHGs, in order to prevent and treat mental health problems and increase mental wellbeing, as this has been proven useful [40]. Second, preventive peer counselling and education regarding human rights should be given to those at risk, which might be easier identified with the help of the factors reported in this study [41]. Third, health-care providers should be trained to recognize depression and other mental health problems, to improve detection of people who need mental health care. Fourth, more collaboration and better coordination is needed between different health-care partners, such as mental health services and the leprosy control programme executed by the basic health services. Finally, this study indicates that for future proposed interventions it is important to take the gender and cultural background of those affected into consideration and highlights the demand for further research on specific interventions to improve the mental health of leprosy-affected people.

## Paper context

Leprosy is an infectious disease that is still one of the most important causes of preventable disability. Next to disability, many leprosy-affected people suffer from stigma and mental health problems.

This study shows that leprosy-affected people had lower mental wellbeing and a higher level of depression than people in the general population. Both mental wellbeing and depression were affected by the level of stigma, gender and disability grade. Therefore, mental health-care interventions are needed.
